# Expression and Prognostic Characteristics of m6A RNA Methylation Regulators in Colon Cancer

**DOI:** 10.3390/ijms22042134

**Published:** 2021-02-21

**Authors:** Liting Huang, Jie Zhu, Weikaixin Kong, Peifeng Li, Sujie Zhu

**Affiliations:** 1 Institute of Translational Medicine, College of Medicine, Qingdao University, Qingdao 266021, China; huangliting15@126.com; 2 Peking University Health Science Center, Department of Pharmacology, School of Basic Medical Sciences, Peking University, 38 Xueyuan Road, Haidian District, Beijing 100191, China; Downsizing@163.com (J.Z.); 1510307407@pku.edu.cn (W.K.)

**Keywords:** colon cancer, m6A regulators, immunity, prognosis

## Abstract

Colon cancer is a common and leading cause of death and malignancy worldwide. N6-methylation of adenosine (m6A) is the most common reversible mRNA modification in eukaryotes, and it plays a crucial role in various biological functions in vivo. Dysregulated expression and genetic changes of m6A regulators have been correlated with tumorigenesis, cancer cell proliferation, tumor microenvironment, and prognosis in cancers. This study used RNA-seq and colon cancer clinical data to explore the relationship between N6-methylation and colon cancer. Based on the seven m6A regulators related to prognosis, three molecular subgroups of colon cancer were identified. Surprisingly, we found that each subgroup had unique survival characteristics. We then identified three subtypes of tumors based on 299 m6A phenotype-related genes, and one subtype was characterized as an immunosuppressive tumor and patients in this subtype may be more suitable for immunotherapy than other subtypes. Finally, using m6A-related genes and clinical information from The Cancer Genome Atlas cohort, we constructed a prognosis model, and this model could be used to predict the prognosis of patients in clinics.

## 1. Introduction

Colon cancer is one of the major morbidities and mortal diseases globally. Cancer Statistics 2020 reported that approximately 140,000 new cases were diagnosed as colon cancer, and there were 53,000 deaths, in 2020 in the USA [1]. Currently, the guidelines for patient stratification and clinical decision remain the use of the AJCC staging system and histologic classification [2]. However, due to colon cancer having a high degree of heterogeneity, prognoses may vary widely between patients with similar clinical features. Therefore, to stratify patients more precisely, it is necessary to go beyond clinical factors. At present, there are two main types of prognostic markers for colon cancer for predicting the prognosis of patients in clinics. The first type is non-invasive, such as liquid biopsy, imaging (PET-MRI) and nanotechnologies. The second type of biomarker related to survival comprises those found in biopsies, such as mast cells (MCs), microRNAs (miRNAs), *KRAS* and v-raf murine sarcoma viral oncogene homologue B (*BRAF*) [3,4]. With the development of transcriptomics, more biomarkers have been discovered.

N6-methylation of adenosine (m6A), which is widely found in mRNA, lncRNA, and miRNA, is the most common reversible mRNA modification in eukaryotes [5,6]. m6A modification is regulated by methyltransferases, demethylases, and binding proteins (also known as “writers”, “erasers”, and “readers”) [7]. m6A is catalyzed by a methyltransferase complex consisting of METTL3, METTL14, WTAP, KIAA1429, ZC3H13, and RBM15, and its demethylation is catalyzed by two “eraser” demethylases, fat mass and obesity-associated protein (FTO) and AlkB homolog 5 (ALKBH5). YTHDF1/2/3, YTHDC1/2, HNRNPA2B1, LRPPRC, and FMR1 are m6A “readers” that can recognize the m6A motif and affect pre-mRNA splicing, transport, stability, and translation [8,9,10]. It has been reported that m6A regulators play a crucial role in various biological functions in vivo. Increasing evidence indicates that dysregulated expression and genetics of m6A regulators is associated with tumorigenesis, cancer cell proliferation, tumor microenvironment (TME), and prognosis in cancers such as glioblastoma, lung cancer, liver cancer, and breast cancer [11,12,13,14,15]. Moreover, CNVs and mutations to the m6A regulator have been related to the prognosis and inferred immune function in Colorectal Cancer (CRC) [16]. However, the biological function and critical target genes of these m6A regulators remain unknown for most human cancers.

Immune processes play critical roles in the carcinogenesis and progression of solid tumors. Several studies have recently revealed a striking correlation between TME-infiltrating lymphocytes and m6A modifications [17]. It has been reported that the interaction of YTHDF1 with the transcripts encoding lysosomal proteases modified by m6A methylation improves the translational efficiency of lysosomal cathepsin in dendritic cells (DCs). The increase of cathepsin in DCs significantly decreases its ability to cross-present tumor antigens, in turn weakening the tumor-infiltrating CD8+ T cell antitumor response [18]. PD1 signaling contributes to a suppressive effect on T cells, and the ligands CD86/CD80 can act as co-stimulatory or co-inhibitory, depending on their binding to CD28 or CTLA4, respectively. PD1 signaling contributes to the suppressive effect on T cells, and the ligands CD86/CD80 can act as co-stimulatory or co-inhibitory, depending on their binding to CD28 or CTLA4, respectively [19]. Upregulated expression of these checkpoint molecules in tumors or stromal cells leads to immunosuppressive TME. Immunotherapy agents for PD1 and CTLA4 have excellent therapeutic benefits in several cancers. However, only a small proportion of patients are sensitive to immunotherapy, so the primary problem is how to select which patients will have an effective response to immunotherapy. Therefore, specific biomarkers or clustering methods are urgently needed to separate responders from nonresponders.

This study aims to investigate the role of m6A regulators in colon cancer from the Gene Expression Omnibus (GEO) and The Cancer Genome Atlas (TCGA) cohort and evaluate the m6A modification pattern with respect to the TME.

## 2. Results

### 2.1. The Landscape of Genetic Variation of m6A Regulators in Colon Cancer

**Figure 1:**
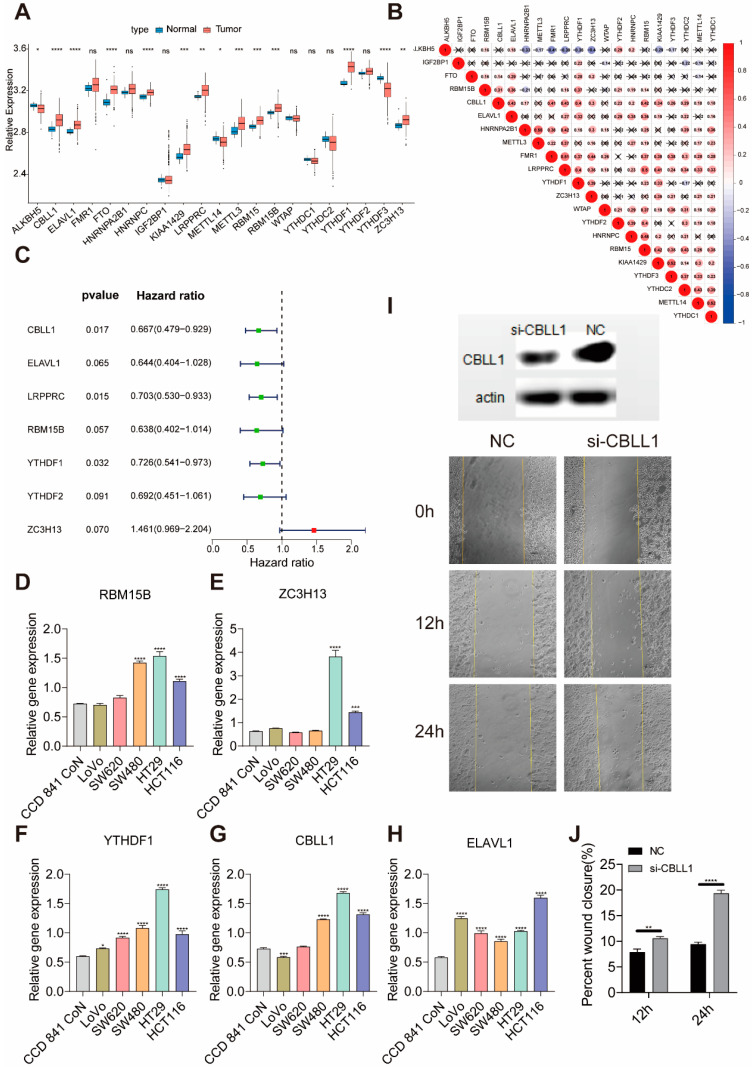
The expression of m6A regulators in colon cancer. (**A**) The expression of 21 m6A regulators between normal tissues and colon cancer tissues. Tumor, red; normal, blue. The upper and lower ends of the boxes represent the interquartile range of values. The lines in the boxes represent the median value, and the black dots show outliers. The asterisks represent the statistical *p*-value (* *p* < 0.05, ** *p* < 0.01, *** *p* < 0.001, **** *p* < 0.0001). (**B**) The interaction between m6A regulators in colon cancer. (**C**) The prognostic analysis for m6A regulators in colon cancer using a univariable Cox regression model. Hazard ratio >1 represents risk factors for survival and hazard ratio <1 represents protective factors for survival. (**D**–**H**) The expression of ELAVL1, RBM15B, ZC3H13, YTHDF1 and CBLL1 in different colon cell lines. (**I**) Western blot analysis of CBLL1 expression in HT29 cells treated for 72 h with CBLL1 siRNA(si-CBLL1) and negative control siRNA (NC), and knockdown CBLL1 promoted wound healing in HT29 cells (20× magnification) by scratch analysis in 0, 12 and 24 h. (**J**) Monitoring of cell migration showed that silencing CBLL1 expression by siRNA promoted migration ability in HT29 cells.

A total of 21 m6A regulators, including eight writers, two erasers, and 11 readers, were identified in this study. Figure 1A shows the mRNA expression levels of regulators between normal and colon cancer samples in the Cancer Genome Atlas (TCGA) database. We found tumors with a high expression of *CBLL1*, *ELAVL1*, *FTO*, *HNRNPC*, *KIAA1429*, *LRPPRC*, *METTL3*, *RBM15*, *RBM15B*, *YTHDF1*, and *ZC3H13*; we also found tumors with a low expression of *ALKBH5*, *METTL3*, and *YTHDF3*. It was found that tumors with a high expression of reader genes (*LRPPRC*, *FMR1*, and *YTHDF1*) showed a low expression of the *ALKBH5* eraser genes, while the high expression of *LRPPRC* and *FMR1* did not affect the expression of another eraser gene (*FTO*). Tumors with high expression of *LRPPRC* showed high expression of *CBLL1*, *FMR1*, *RBM15*, *KIAA1429*, *YTHDF1*, and low expression of *ALKBH5* (Figure 1B). A univariate Cox regression model revealed 21 m6A regulators’ prognostic values in patients with colon cancer, in which seven genes (*CBLL1*, *ELAVL1*, *LRPPRC*, *RBM15B*, *YTHDF1*, *YTHDF2*, and *ZC3H13*) were associated with prognosis (Figure 1C). Next, we explored the expression of *ELAVL1*, *RBM15B*, *YTHDF1*, *CBLL1*, and *ZC3H13* in normal colon cells and different kinds of colon cancer cell lines. We found that the five m6A regulators vary in different colon cancer cells (Figure 1D,H). These genes are highly expressed in colon cancer cells in situ (HT29 and HCT116 colon cancer cell lines) and show a relatively low expression in cell lines with high metastatic characteristics (LoVo and SW620 cell lines). Thus, we hypothesized that the expression of these genes might be related to the tumor cells’ ability to metastasize. Among them, the expression trend of *CBLL1* was consistent with the ability of cell metastasis, so we selected *CBLL1* for further verification. HT29 cells were selected for knock-out of the *CBLL1* gene, and cell metastasis was verified using a scratch test. According to the experimental results, abolishing the expression of the *CBLL1* gene can increase the metastasis of tumor cells HT29 (Figure 1I–J). This may explain why patients with high *CBLL1* expression have relatively better survival.

### 2.2. Construction of Three Molecular Subgroups of Colon Cancer Using Seven m6A Regulator with Prognosis

**Figure 2:**
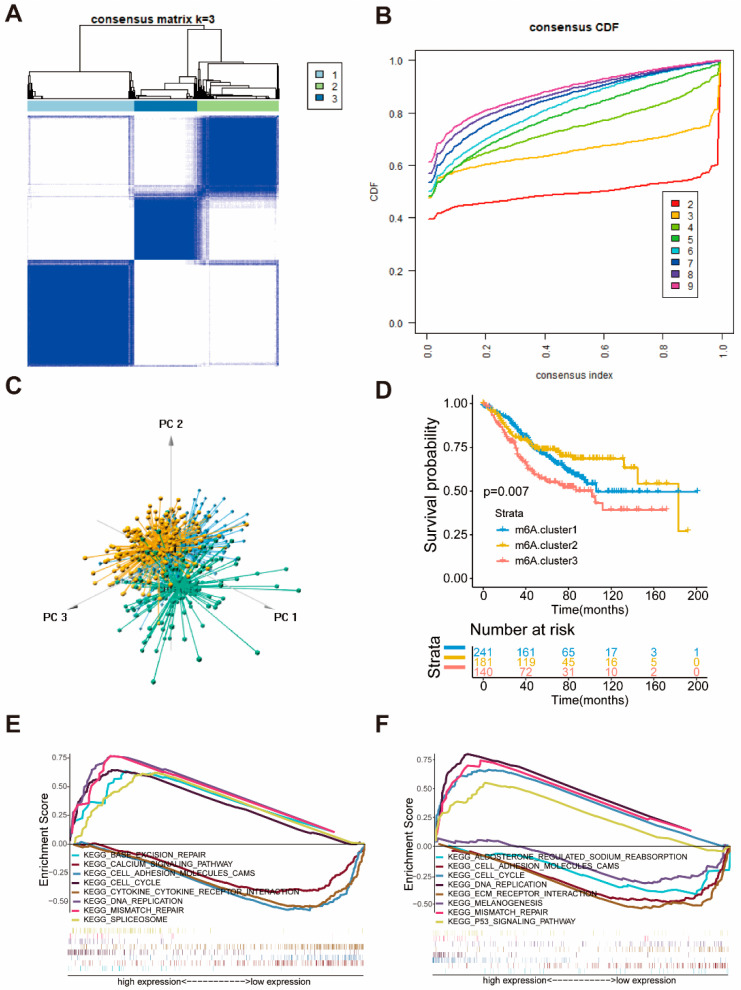
Identification of three molecular subgroups of colon cancer by seven m6A regulators associated with prognosis and biological characteristic of each subgroups. (**A**) The consensus matrices of the GSE39582 for k = 3. (**B**) The cumulative function (CDF) curves in consensus cluster analysis. CDF curves of consensus score by different subtype number (k = 2–9) are represented. (**C**) Three-dimensional scaling plot by transcriptome profile of three subgroups. (**D**) Survival analyses of three subgroups based on 566 patients with colon cancer from GEO cohorts (GSE39582), including 241 cases in m6A.cluster1, 181 cases in m6A.cluster2, 140 cases in m6A.cluster3. Kaplan-Meier curves with Log-rank *p*-value 0.007 showed a significant survival difference among the three m6A modifications. m6A.cluster3 showed a poorer overall survival than the other two m6A.clusters. (**E**,**F**) GSEA enrichment analysis showing the activation states of biological pathways in three molecular subgroups of colon cancer. (**E**) m6A.cluster1 vs. m6A.cluster3. (**F**) m6A.cluster2 vs. m6A.cluster3.

According to the expression of seven m6A regulators associated with prognosis, patients were classified into qualitatively different m6A modification patterns using the R software package of ConsensesClusterPlus, and three molecular subgroups were eventually identified using unsupervised clustering, including 241 cases in m6A.cluster 1, 181 cases in m6A.cluster 2, and 140 cases in m6A.cluster 3, respectively (Figure 2A–C). Comparison of prognosis of three major m6A modified subtypes revealed a particularly prominent survival advantage in m6A.cluster1/2 (Figure 2D). 

To investigate the biological behavior among different molecular subgroups, we constructed Gene Set Enrichment Analysis (GSEA). Figure 2E shows that the m6A.cluster1 was markedly enriched in base excision repair, cell cycle, DNA replication, and mismatch repair signaling pathway. The activation of these signaling pathways means that the patients in cluster1 may have stronger DNA repair capabilities. The m6A.cluster2 presented enrichment in pathways associated with the P53 signaling pathway, DNA replication, cell cycle, and mismatch repair signaling pathway. In comparison, the m6A.cluster3 was prominently related to melanogenesis, ECM receptor interaction, cell adhesion, and cytokine-cytokine receptor interaction signaling pathway (Figure 2F). Activation of these signaling pathways in cluster3 may be conducive to metastasis of cancer.

### 2.3. The Characteristics of the Three Molecular Subgroups of Colon Cancer

**Figure 3:**
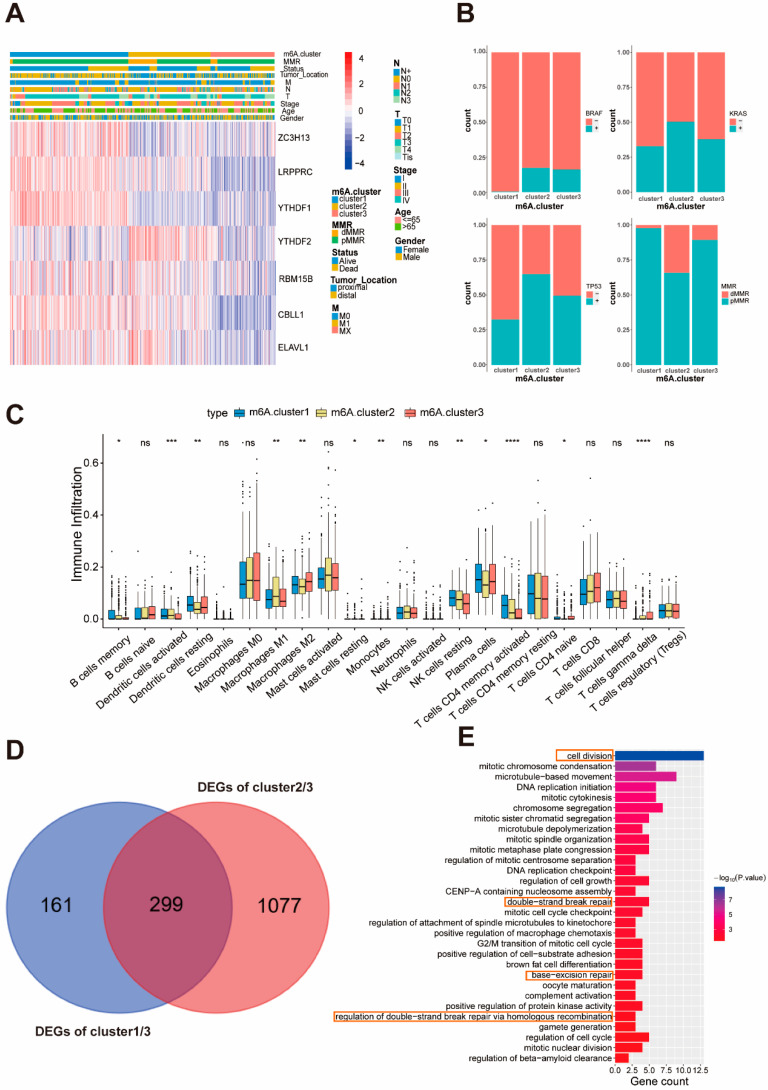
Immune cells and clinical characteristics in the three molecular subgroups. (**A**) Unsupervised clustering of seven m6A regulators in the GSE39582. The m6A.cluster, stage, status, M, N, T, age and gender were used as patient annotation. Red represents high expression of regulators and blue represents low expression. (**B**) Difference in the clinical features including BRAF/KRAS/TP53 mutant and MMR status among three m6A.cluster. (**C**) The abundance of each immune cells in the three m6A.clusters. The upper and lower ends of the boxes represent the interquartile range of values. The lines in the boxes represent the median value, and the black dots show the outliers. The asterisks represent the statistical *p*-value (* *p* < 0.05, ** *p* < 0.01, *** *p* < 0.001, **** *p* < 0.0001). (**D**) 299 m6A-related phenotype-related genes shown in Venn diagram. (**E**) Functional annotation for m6A-related genes using GO enrichment analysis. The orange boxes represent signaling pathways involved in DNA repair and cell division.

A significant distinction existed in the m6A transcriptional profile among the three molecular subgroups of colon cancer (Figure 2C). m6A.cluster1 was characterized by increased expression of *ZC3H13*, *LRPPRC*, *YTHDF1* and *CBLL1*, and presented a small decrease in *YTHDF2*. We also noted that tumors with m6A.cluster3 had a lower expression of *ZC3H13*, *LRPPRC*, *YTHDF1*, *YTHDF2*, *RBM15B*, *CBLL1*, and *ELAVL1* (Figure 3A). Next, we analyzed the distribution of factors commonly used for colon cancer prognosis in these three subgroups. As the results indicated (Figure 2B), the m6A.cluster1 has a low number of BRAF/KRAS/TP53 mutations, which is typically associated with survival [20]. Previous studies have demonstrated that patients with dMMR have a prominent survival advantage in colon cancer [21], which is consistent with the analysis. According to previous studies, M6A modification is related to the immune response and accumulation of immune cells [21,22]. We then used a deconvolution algorithm based on support vector regression, the CIBERSORT method, to determine the type of immune cells in the tumor [23], and compared the component differences in immune cells among the three clusters. In previous studies, the densities of CD4 + T cells in pre-treatment biopsies predicted a favorable response to therapy, a finding that has been widely used for developing immunotherapy [24]. Tumor-associated macrophages (TAMs) include both M1 macrophages and M2 macrophages. M1 macrophages are involved in promoting anti-tumor immunity, but M2 macrophages possess pro-tumorigenic properties [25,26]. To our surprise, the m6A.cluster1 was remarkably rich in CD4+ T cells, NK cells, and dendritic cells (Figure 3C), responsible for antigen presentation and the activation of naive T cells [24,27], indicating that the m6A.cluster1 has an anti-tumor immune status. Compared with cluster1, m6A.cluster3 has fewer CD4 + T cells and NK cells, and more M2 cells, and thus it may be associated with a state of immunosuppression. As we know, the high expression of the immune checkpoint genes is another characteristic of immunosuppression [28,29,30]. Thus, we examined the expression of these genes in three clusters (Figure S2A). Consistent with the previous analysis, the expression of the immune checkpoint in cluster3 is the highest within these three clusters, indicating that patients in m6A.cluster3 are more likely to have an immune escape. These results seem to reveal this typing with genes related to m6A, possibly highlighting a new factor that can be used in immunotherapy for patient screening.

### 2.4. Generation of m6A Phenotype Genes and Function

**Figure 4:**
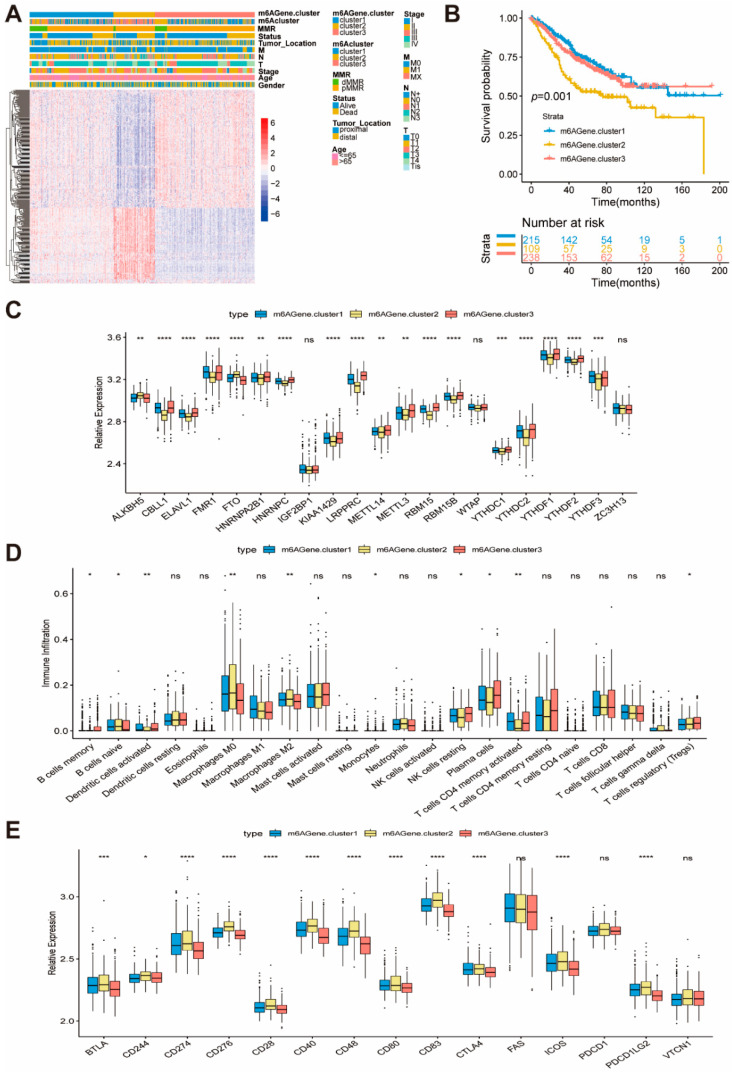
Construction of m6A phenotype-related genes and function. (**A**) Unsupervised clustering of overlapping m6A phenotype-related genes in GSE39582 to classify patients into different genomic subtypes, termed m6AGene.cluster1/2/3, respectively. The m6AGene.cluster, m6Acluster, MMR, status, tumor_location, M, N, T, stage, age and gender were used as patient annotations. (**B**) Kaplan-Meier curves indicated m6A modification genomic phenotype were markedly related to overall of 562 patients in GSE39582, of which 215 cases were in m6Agene.cluster1, 109 cases were in m6Agene.cluster2, 238 cases were in m6Agene.cluster3. (*p* = 0.001). (**C**) The expression of 21 m6A regulators in the three gene clusters. The upper and lower ends of the boxes represent the interquartile range of values. The lines in the boxes represent the median value, and the black dots show the outliers. The asterisks represent the statistical *p*-value (* *p* < 0.05, ** *p* < 0.01, *** *p* < 0.001, **** *p* < 0.0001). (**D**) The abundance of each immune cells in the three gene clusters. The upper and lower ends of the boxes represent the interquartile range of values. The lines in the boxes represent the median value, and the black dots show the outliers. The asterisks represent the statistical *p*-value (* *p* < 0.05, ** *p* < 0.01, *** *p* < 0.001, **** *p* < 0.0001). (**E**) The expression of checkpoint genes in the three gene clusters. The upper and lower ends of the boxes represent the interquartile range of values. The lines in the boxes represent the median value, and the black dots show the outliers. The asterisks represent the statistical *p*-value (* *p* < 0.05, ** *p* < 0.01, *** *p* < 0.001, **** *p* < 0.0001).

To further investigate each molecular subgroup’s potential biological behavior, we determined 299 subgroups of colon cancer-related differentially expressed genes (DEGs) using the limma package (Figure 3D). The clusterProfilter package was used to perform GO enrichment analysis for DEGs. Surprisingly, these genes showed enrichment of biological processes related to DNA repair and cell division (Figure 3E). Moreover, KEGG enrichment analysis for signaling pathways (Figure S2B) was remarkably associated with p53 signaling pathway, ECM receptor, and infection signaling pathways. Given that three subgroups of colon cancer are identified by the m6A regulator, we named the 299 genes m6A phenotype genes and we confirmed that the m6A phenotype genes plays a non-negligible role in DNA repair and TME in the tumor (Figure 3E and Figure S2B). Then, based on the 299 obtained m6A phenotype-related genes, unsupervised clustering analyses were performed to further validate this regulation mechanism and classify patients into different genomic subtypes, which we named m6AGene cluster1–3. Opposite patterns were observed in the m6AGene cluster2 and cluster3 (Figure 4A). A total of 215 and 238 patients with colon cancer were clustered in m6AGene cluster1 and cluster3, respectively, which were proved to have better prognosis. In contrast, patients in m6AGene cluster2 (109) experienced a poorer prognosis (Figure 4B). Analysis of the composition of immune cells in these clusters indicated that m6AGene.cluster2 has fewer CD4 + T cells, plasma cells, and NK cells, and more M0/M2 macrophages, which indicates a poorer anti-tumor environment (Figure 4D). We also investigated the expression of immune checkpoint genes. As shown in Figure 4E, most of the checkpoint genes were upregulated in m6AGene.cluster2. Combining these data, we hypothesize that the patients in m6AGene.cluster2 are in a state of tumor immune escape. Due to immunosuppression, the m6AGene.cluster2 had poorer survival. In the three m6AGene.clusters, prominent differences in the expression of m6A regulators were observed (Figure 4C). We observed a lower expression of *LRPPRC*, *CBLL1*, and high expression of *ALKBH5* in the m6AGene.cluster2, which has a poorer prognosis. A survival benefit trend was observed in patients with low *ALKBH5* and high *LRPPRC* and *CBLL1* (*GSE38832*, Figure S3C). Next, we explored the relationship between *ALKBH5*, *LRPPRC*, and *CBLL1* genes, and clinical factors. With the T stage’s aggravation, the expression of *LRPPRC* increased, which is consistent with previous results indicating that the patients with higher *LRPPRC* expression had a poorer survival. Moreover, the *BRAF* wild-type group had a high expression of *LRPPRC* and *CBLL1* (Figure S4A,B). *CBLL1* was correlated with the N stage, combined with the qPCR results showing that low CBLL1 expression in highly metastatic cell lines (Figure 1H), indicates that *CBLL1* is related to colon tumor metastasis, while *ALKBH5* is associated with T and N stages (Figure S4B,C). The above results show that m6A methylation modification regulator plays a non-negligible regulation role in shaping different TME-infiltrated immune cells.

### 2.5. Establishment of the Prognostic Model

**Figure 5:**
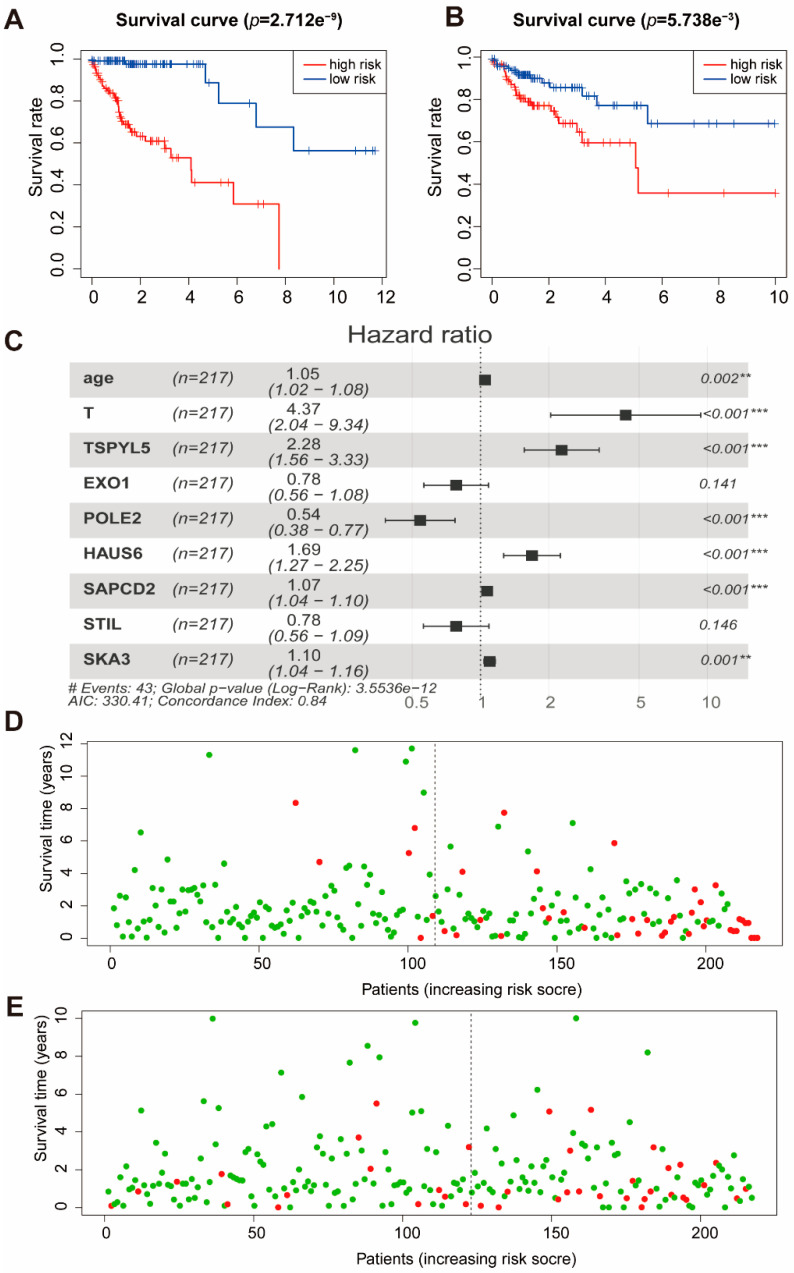
Cox regression model results in TCGA. (**A**) The survival curves for the m6A risk score in the training set. Grouping was based on the median m6A risk score in the training set. Red is the high-level group, and blue is the low-level group. (**B**) The survival curves for the m6A risk score in the test set. Red is the high-level group, and blue is the low-level group. Grouping was based on the median m6A risk score in the training set. (**C**) A forest plot of the multivariate Cox regression model. Hazard ratio is provided in the figure. ** *p* < 0.01, *** *p* < 0.001. (**D**) Patient survival status in the training set. The x-axis is the patient ranking in ascending order by m6A risk score; the y-axis is survival time. The red dots are the dead patients, and the green dots are the surviving patients. (**E**) Patient survival status in the test set. The x-axis is the patient ranking in ascending order by m6A risk score; the y-axis is survival time. The red dots are the dead patients, and the green dots are the surviving patients.

Considering the importance of clinical features in cancer, we constructed a prognostic model based on these m6A phenotype-related genes (299) and five clinical features. Four hundred and thirty-four patients were divided into a training set (*n* = 217) and a test set (*n* = 217). The clinical data and grouping of patients are shown in Table 1. To illustrate the random grouping’s rationality, differences in clinical variables between the training set and the test set were examined. First, Lasso regression was performed for 299 genes and five clinical features to eliminate collinearity between the variables. Sixteen variables were used to establish a multivariate Cox regression model, and the independent variables were selected by back-off method. Finally, nine variables were included in the Cox regression model (Figure 5C). The model contains clinical information and genes related to the m6A regulator, and we call this model the m6A risk score. The formula of the m6A risk score was as follows:

m6A risk score = 0.045123*Age + 1.473787*T + 0.823294*TSPYL5 – 0.24659*EXO1 – 0.61275*POLE2 + 0.522568*HAUS6 + 0.067226*SAPCD2 – 0.24671*STIL + 0.093139*SKA3.

Age is given in years; T is an integer from 1 to 4; the levels of the four genes are the normalized values of FPKM (Fragments Per Kilobase of exon model per Million mapped fragments).

According to the median value (1.17) of the m6A risk score in the training set, the training and test sets were grouped, patients with an m6A risk score of less than 1.17 were in the low-risk group, and m6A risk score greater than 1.17 were in the high-risk group. Moreover, Kaplan-Meier curves were used for survival analysis. We found that those with high m6A risk scores had lower survival time in both the training set (Figure 5A) and the test set (Figure 5B). The patients were classified according to the m6A risk score, and the distribution of patient survival status was plotted. In both the training set and the test set, patient survival time gradually decreased, and the number of deaths gradually increased with the increase in m6A risk score (Figure 5D,E). We used the m6A risk score in the training set and the test set to predict patient survival status at 1, 3, and 5 years, and plotted the receiver operator characteristic curves. The area under the curve (AUC) of the m6A risk score in the training set for 1, 3, and 5 years was 0.852, 0.874, and 0.795, respectively (Figure S5C). The AUC of the m6A risk score in the test set for 1, 3, and 5 years was 0.64, 0.683, and 0.678, respectively (Figure S5D). The above results indicate that the prognostic model based on m6A-related genes and clinical information was effective.

### 2.6. Verification of m6A Risk Score

**Figure 6:**
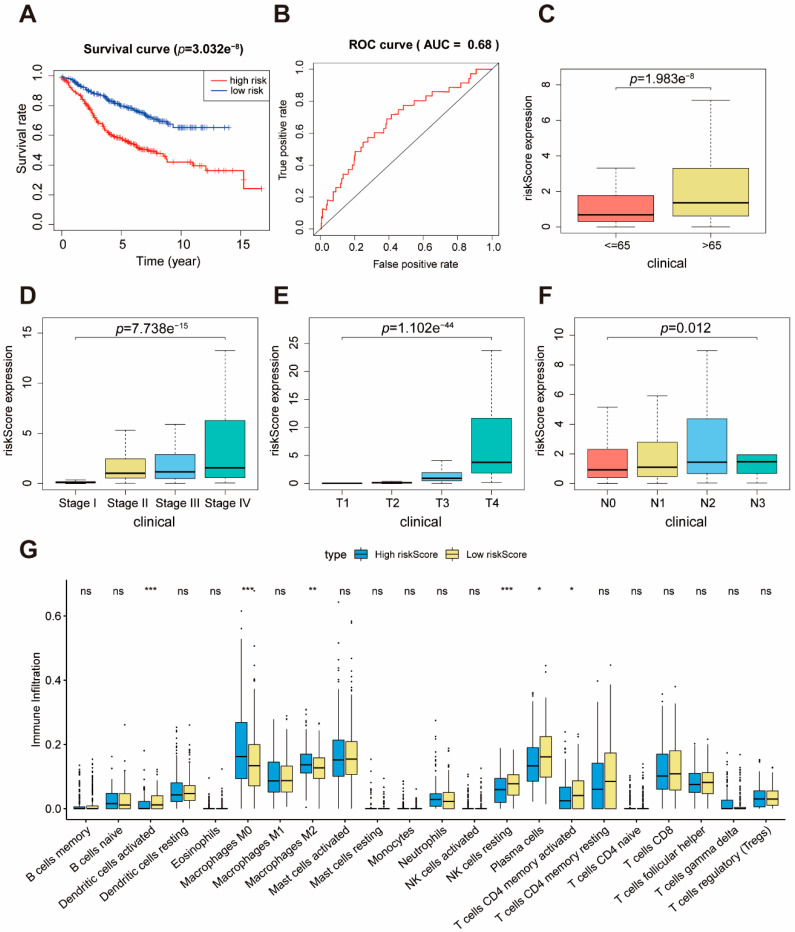
Validation of the prognostic performance of m6A risk score in GSE39582. (**A**) The survival curves of the m6A risk score in GSE39582. Grouping was based on the median m6A risk score in the training set. Red is the high-level group, and blue is the low-level group. (**B**) ROC curve of the m6A risk score forecast result after 1 year. (**C**–**F**) The relationship between m6A risk score and clinical features of patients. (**G**) Estimated immune cell expression in high and low m6A risk score groups.

To further verify our prognostic model’s effectiveness, we applied the m6A risk score established in the TCGA cohort to other independent colon cancer cohorts to verify its prognostic value. The ability of the m6A risk score to predict relapse-free survival was also evaluated in GEO database (GSE39582). Consistent with the results in TCGA cohort, patients with high risk scores had poorer survival than the patients with low risk scores (Figure 6A). The survival time of patients gradually decreased, and the number of deaths gradually increased, with the increase in m6A risk score (Figure S6B). The AUC of the m6A risk score in the GSE39582 set for 1, 3, and 5 years was 0.68, 0.663, and 0.632, respectively (Figure 5B and Figure S6C,D). Furthermore, we explored whether the clinical factors (Age/Stage/T/N) strongly correlated with the m6A risk score. Hence, the Wilcox and Kruskal test was performed in the GEO set. We observed that the higher the m6A risk score, the poorer the patient’s condition, demonstrating that the m6A risk score can predict patient survival (Figure 6C–F). We also noted that a low m6A risk score had more CD4+ T cells, plasma cells, NK cells, DCs, and fewer M0/M2 macrophages (Figure 6G). These findings provide evidence supporting the conclusion that m6A risk score is closely related to survival and immunity.

## 3. Discussion

Similar to reversible epigenetic modifications such as DNA methylation, m6A RNA modifications can be added by writer enzymes and removed by eraser enzymes. Differential expression of specific RNA m6A regulators is associated with abnormally regulated RNA in tumors. However, the same m6A methylation regulator may play distinct roles in different tumors. Here, three molecular subgroups of colon cancer with significantly distinct characteristics were revealed, based on seven m6A regulators associated with prognosis. Compared with the other clusters, m6A.cluster1 was characterized by a lower number of *BRAF*/*TP53*/*KRAS* mutations, more CD4+ T cells/plasma cells/DC cells, and low expression of checkpoint genes, corresponding to a strongly immune-activated and repaired status. m6A.cluster2 was characterized by activation of the p53 pathway, more *BRAF*/*TP53*/*KRAS* mutants, more CD4+ T cells, and moderate levels of immune checkpoint expression, corresponding to moderate immune activation. Finally, m6A.cluster3 was characterized by activation of ECM receptor interaction, cell adhesion, cytokine-cytokine receptor interaction, fewer CD4+ T cells/plasma cells/NK cells, more M2 cells, and high expression of checkpoint genes, corresponding to immune-suppression phenotypes. We know that immune-suppression phenotypes are associated with immune tolerance and ignorance, and lack of activated and priming T cells [31].

Furthermore, in this study, the mRNA transcriptome differences between distinct subgroups of colon cancer were found to be significantly correlated with cell adhesion, ECM receptor, and DNA replication-related biological pathways. The DEGs were denoted as m6A phenotype-related genes (299). Similar to the m6A modification regulator clustering results, three genomic subtypes were identified on the basis of m6A phenotype-related genes (299) that were also significantly correlated with immunity. We also analyzed the immune cell composition of three genomic subtypes of colon cancer based on m6A phenotype-related genes (299). We found that m6AGene.cluster2, which has poorer survival, had lower numbers of CD4 + T cells, more M2 cells, and high expression of checkpoint genes, among the three m6AGene.cluster. This work showed that m6A methylation modification regulator played a non-negligible role in immunity, and patients in m6AGene.cluster2 may be better candidates for immunotherapy. We also observed that m6AGene.cluster2 had low expression of *LRPPRC*, *CBLL1*, and high expression of *ALKBH5*, given that we analyzed the prognosis associated with the three genes. We found that patients with low expression of *ALKBH5* and high expression of *LRPPRC* and *CBLL1* had increased survival, which may be correlated with the T or N stages and BRAF status. Next, we proposed exploring the feasibility of the common 299 m6A phenotype-related genes in colon cancer prognosis estimation and then constructed a prognostic model with these related genes. The prognostic model m6A risk score built in this study provides a reference for treating patients with colon cancer. 

In this model, low age and low T stages were favorable factors for prognosis. *TSPYL5* (TSPY-like 5, also known as KIAA1750) is a member of the testis-specific protein Y-encoded-like (TSPY-L) family of genes. The *TSPYL5* gene is frequently amplified in breast cancer [32,33]. The TSPYL5 was detected in 21–27% of primary breast tumor samples and is associated with a higher propensity of metastatic recurrence. Furthermore, TSPYL5 is a suppressor of p53 function through its interaction with USP7, and the promotion of P53 degradation by TSPY1 influences the activity of P53 target molecules (CDK1, P21, and BAX) to expedite the G2/M phase transition and decrease cell apoptosis, accelerating cell proliferation [34,35]. In our prognosis model, *TSPYL5* expression is also associated with a poor outcome. POLE2 is the DNA polymerase epsilon B subunit and, accordingly, participates in DNA replication, repair, and cell cycle control functions [36,37], which is included in the array-based proliferation signature [38]. The HAUS6 (HAUS augmin-like complex subunit 6) is a subunit of the augmin complex that affects microtubule attachment to the kinetochore and central spindle formation, possibly having a role in efficient chromosome congression and segregation by promoting microtubule-dependent microtubule amplification. *SAPCD2* (Suppressor anaphase-promoting complex domain containing 2) plays important roles in the initiation, invasion, and metastasis of several malignancies. Previous studies have shown that *SAPCD2* expression was significantly higher in colon cancer tissues than in adenoma and normal epithelial tissues, and it significantly promoted cell proliferation, migration, and invasion both in vitro and in vivo [39]. SKA3 (spindle- and kinetochore-related complex subunit 3) is a component of the spindle- and kinetochore-related complexes and is essential for accurate timing of late mitosis. Hepatocellular carcinoma patients with high levels of SKA3 expression have a poor prognosis. SKA3 was found to affect tumor progression through the cell cycle and the P53 signaling pathway [40].

In short, the m6A risk score could be used in clinical practice to guide more effective clinical practice. We also demonstrated that the m6A risk score could be used to assess clinical tumor stages and tumor inflammation. We found a complex relationship between the m6A risk score and clinicopathological features. However, some limitations of this study should be noted. Although an independent validation set validated the m6A risk score, its predictive ability for clinical research remains unclear. Moreover, this study is a bioinformatic analysis, and the potential functional mechanisms of the m6A-related genes were not studied. Hence, other cell and animal studies should be performed to elucidate the role of m6A-related genes in colon cancer.

## 4. Materials and Methods

### 4.1. Cell Line, RNA Extraction and qRT-PCR

Human colon cancer cell line LoVo, SW620, SW480, HT29 and HCT116 and human colon epithelial cells CCD841 CoN were obtained from ATCC® Cell Lines (Manassas, VA, USA). The cells were cultured in DMEM medium (Meilunbio, Dalian, China) with 10% fetal bovine serum (Gibco, Thermo Fisher Scientific, Inc., Waltham, MA, USA) at 37 °C with 5% CO2. The identified cell lines were used in this study. Total RNA was extracted from cultured cells, and quantitative real-time PCR was performed using Life Technologies QuantStudio 3 (Thermo Fisher Scientific, Inc., Waltham, MA, USA). The primers used in qRT-PCR are listed in Table S1.

### 4.2. SiRNA Transfection, RNA Interference and Scratch Test

In RNA interference experiments, si-CBLL1 corresponding to the target sequence and control siRNA were purchased from GenePharma (Shanghai, China). The sequences of siRNA used in this experiment are listed in Table S2. All siRNA transfection experiments were performed with lip3000 reagent (Thermo Fisher Scientific, Inc., Waltham, MA, USA, L3000015). After 72 h of transfection, the knockdown efficiency was detected by Western blot. After 24 h of transfection, the cells were seeded into a 24-well plate with a density of 90–100%. After 12 h of culture, the cells were completely adhered to the wall. A cross line was drawn at the bottom of each well with a 20-μL pipette tip. The detached cells were then removed by washing with PBS. The migration of cells to the wound area was recorded at 0, 12 and 24 h.

### 4.3. Colon Cancer Dataset and Preprocessing

The workflow of our study is shown in Figure S1A. Public gene-expression data and full clinical annotation were searched in GEO (http://www.ncbi.nlm.nih.gov/geo/) and TCGA database (https://portal.gdc.cancer.gov/). Patients without survival information were removed from further evaluation. For microarray data from Affymetrix^®^ (Affymetrix, Santa Clara, CA, USA), we downloaded the raw “CEL” files and applied the “limma” package in R for the analysis. As for datasets in TCGA, RNA sequencing data (FPKM value) of gene expression were downloaded from the Genomic Data Commons (GDC, https://portal.gdc.cancer.gov/). The somatic mutation data were acquired from the TCGA database. Data were analyzed with the R (version 3.6.2) and R Bioconductor packages [41].

### 4.4. Unsupervised Clustering for Seven m6A Regulators Related to Prognosis

The seven m6A regulators related to prognosis included CBLL1, ELAVL1, LRPPRC, RBM15B, YTHDF1, YTHDF2, ZC3H13 (Figure 1C). Unsupervised clustering analysis was applied to identify distinct vitiligo modification patterns based on the expression of seven m6A regulators and classify patients for further analysis. We used the Consensus Cluster Plus package (http://www.bioconductor.org/packages/release/bioc/html/ConsensusClusterPlus.html) to perform the above steps and 1000 times repetitions were conducted for guaranteeing the stability of classification [42].

### 4.5. Immune Profiles in Colon Molecular Subtypes

To quantify the proportions of immune cells in the melanoma cancer samples, we used the CIBERSORT algorithm, which allows for sensitive and specific discrimination of 22 human immune cell phenotypes. CIBERSORT is a deconvolution algorithm that uses a set of reference gene-expression values (a signature with 547 genes) considered a minimal representation for each cell type and, based on those values, infers cell-type proportions in data from bulk tumor sample with mixed cell types using support vector regression. The expression value of checkpoint genes is compared between different colon cancer subtypes. One-way ANOVA was used to conduct different comparisons of three groups.

### 4.6. Identification of Differentially Expressed Genes (DEGs) between m6A Distinct Phenotypes

To identify genes associated with m6A-related genes, we grouped patients into m6A.cluster1–3 based on the expression of seven m6A regulators. DEGs among m6A.cluster1 and m6A.cluster3 (m6A cluster2 and m6A cluster3) were determined using the R package limma (http://www.bioconductor.org/packages/release/bioc/html/limma.html), which implements an empirical Bayesian approach to estimate gene-expression changes using the moderated *t*-test. DEGs among colon subtypes were determined by significance criteria (adjusted *p*-value < 0.05) as implemented in the R package limma. The adjusted *p*-value for multiple testing was calculated using the Benjamini-Hochberg correction.

### 4.7. Functional and Pathway Enrichment Analysis

Gene annotation enrichment analysis using the clusterProfiler R package (http://www.bioconductor.org/packages/release/bioc/html/clusterProfiler.html) was performed on m6A-related genes. Gene Ontology (GO) terms were identified with a strict cutoff of *p* < 0.01 and false discovery rate (FDR) of less than 0.05. We also identified the pathway that was up and down regulated among m6A.cluster1 and m6A.cluster3 (m6Acluster2 and m6A.cluster3) by running a gene set enrichment analysis (GSEA, http://software.broadinstitute.org/gsea/index.jsp) of the adjusted expression data for all transcripts. 

### 4.8. Verification of the m6A Risk Score

The samples in the training and test sets were grouped according to the median value of the m6A risk score in the training set, and then Kaplan-Meier survival curves with log-rank test were plotted. The following other verifications were performed on the training and test sets at the same time to verify the validity of the m6A risk score: (1) survival status of patients after 1, 3, and 5 years were predicted, and the receiver operating characteristic (ROC) curves were plotted to calculate the AUC. (2) Wilcox and Kruskal test were used to examine the correlation between m6A risk score and clinical information.

### 4.9. Statistical Analysis

Wilcox and Kruskal-Wallis tests were used to conduct different comparisons of three or more groups. With the median value as cutoff, patients were divided into low or high m6A risk score groups. The survival curves for the prognostic analysis were generated via the Kaplan-Meier method and a log-rank test was used to identify the significance of differences. The forest plot R package (http://ydl.oregonstate.edu/pub/cran/web/packages/forestplot/index.html) was employed to visualize the results of the multivariate prognostic analysis for 21 m6A regulators in the GEO database. All statistical *p*-values were two-sided, with *p* < 0.05 being statistically significant. All data processing was done in R 3.6.2 software (https://cran.r-project.org/bin/windows/base/old/3.6.2/) [41].

## 5. Conclusions

The role of m6A RNA modifications in patients with CRC is arousing concern. Our data show that seven m6A regulators (CBLL1, ELAVL1, LRPPRC, RBM15B, YTHDF1, YTHDF2, and ZC3H13) are related to tumorigenesis, tumor microenvironment and tumor prognosis. Through the study of three subtypes based on 299 m6A phenotype-related genes, one subtype was characterized as immunosuppressive tumors, and patients in this subtype may be more suitable for immunotherapy than other subtypes. Finally, using m6A-related genes and clinical information, a prognosis model was constructed, and may be used to predict the prognosis of CRC patients in clinics.

## Figures and Tables

**Table 1:** Clinical characteristics of colon cancer patients.

Characteristics	TCGA Cohort		GSE39582 (*n* = 531)
	Training Set (*n* = 217)	Test Set (*n* = 217)	
Age (mean)	67.92	66.35	66.77
Gender (%)			
female	101	107	244
male	116	110	287
Stage			
I	29	45	31
II	90	85	251
III	67	57	190
IV	31	30	59
T			
T1	1	8	11
T2	33	40	43
T3	161	137	360
T4	22	32	117
N			
N0	121	136	294
N1	57	43	133
N2	39	48	104

## Data Availability

Publicly available datasets were analyzed in this study. This data can be found here: https://www.cancer.gov/about-nci/organization/ccg/research/structural-genomics/tcga (accessed on 10 January 2021) and https://www.ncbi.nlm.nih.gov/gds/ (accessed on 10 January 2021).

## Supplementary Materials

The following are available online at https://www.mdpi.com/1422-0067/22/4/2134/s1.
