# Supplementary material for: Expression and Prognostic Characteristics of m6A RNA Methylation Regulators in Colon Cancer
Source: Int J Mol Sci. 2021 Feb 21;22(4):2134. doi: 10.3390/ijms22042134 (PMC7926939; doi:10.3390/ijms22042134)
Supplement: Supplementary file 1 [file ijms-22-02134-s001.pdf]

Supplementary Materials

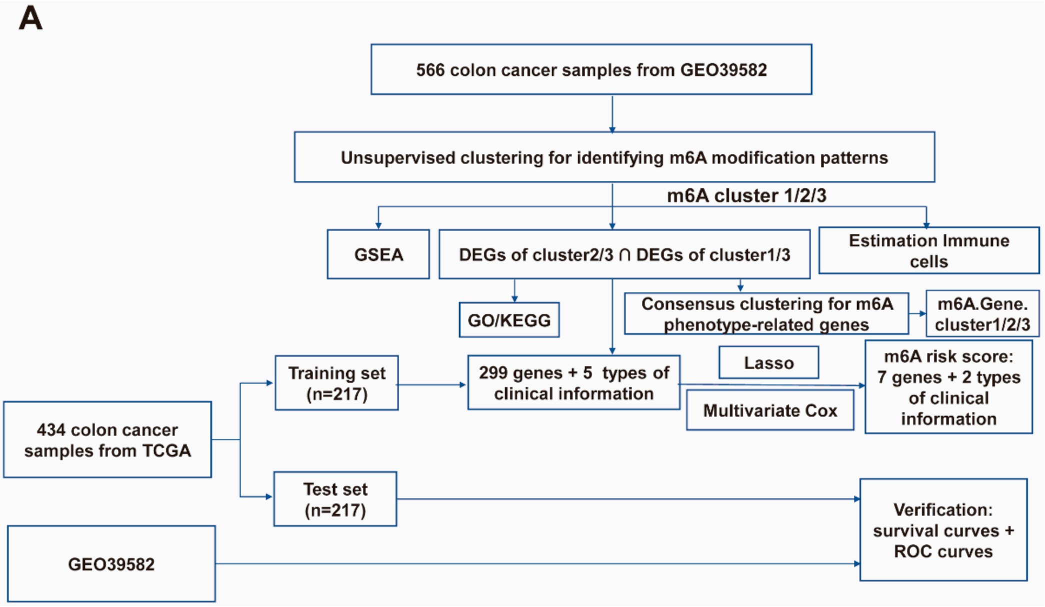

Figure S1. Overview of study design. (A) Overview of this work.

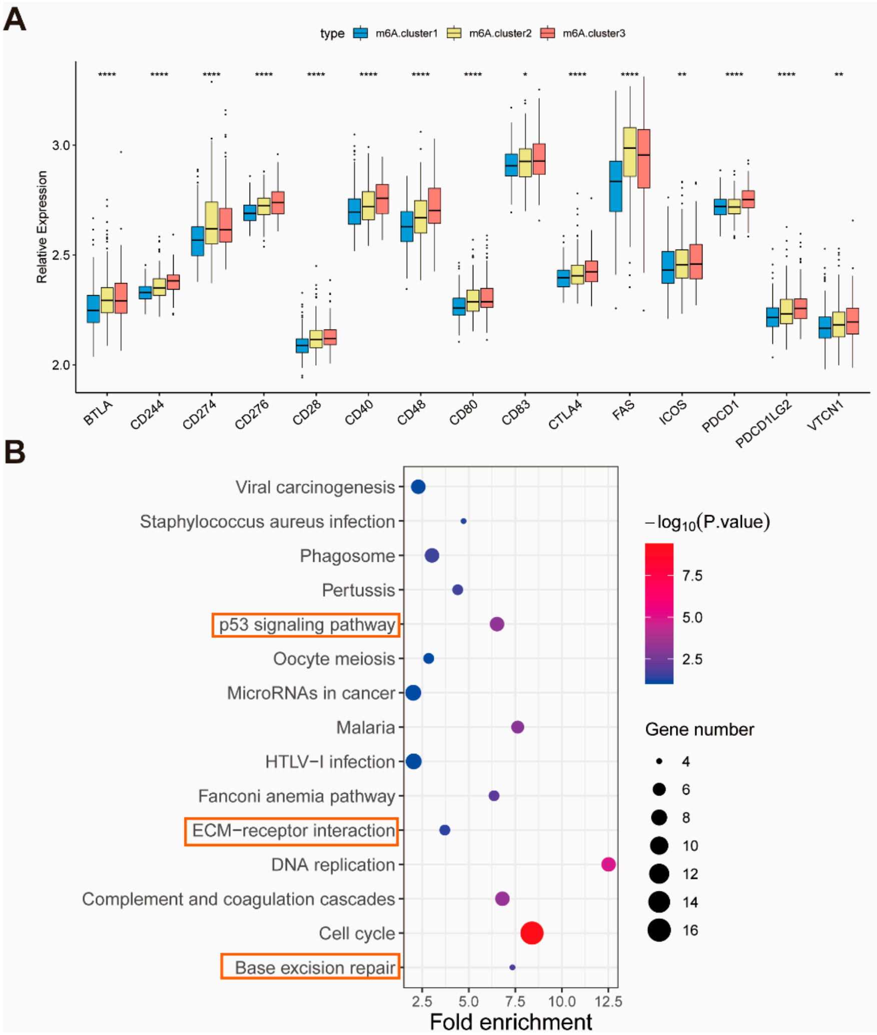

Figure S2. Characteristic of each m6A modification pattern. (**A**) The expression of checkpoint

genes in three m6A modification patterns. The upper and lower ends of the boxes represented interquartile range of values. The lines in the boxes represented median value, and the black dots showed outliers. The asterisks represented the statistical p value (* $p < 0.05$, ** $p < 0.01$, *** $p < 0.001$, **** $p < 0.0001$). (**B**) Signaling pathway for m6A-related genes using KEGG analysis. The orange boxes represent signaling pathways involved in oncogenesis.

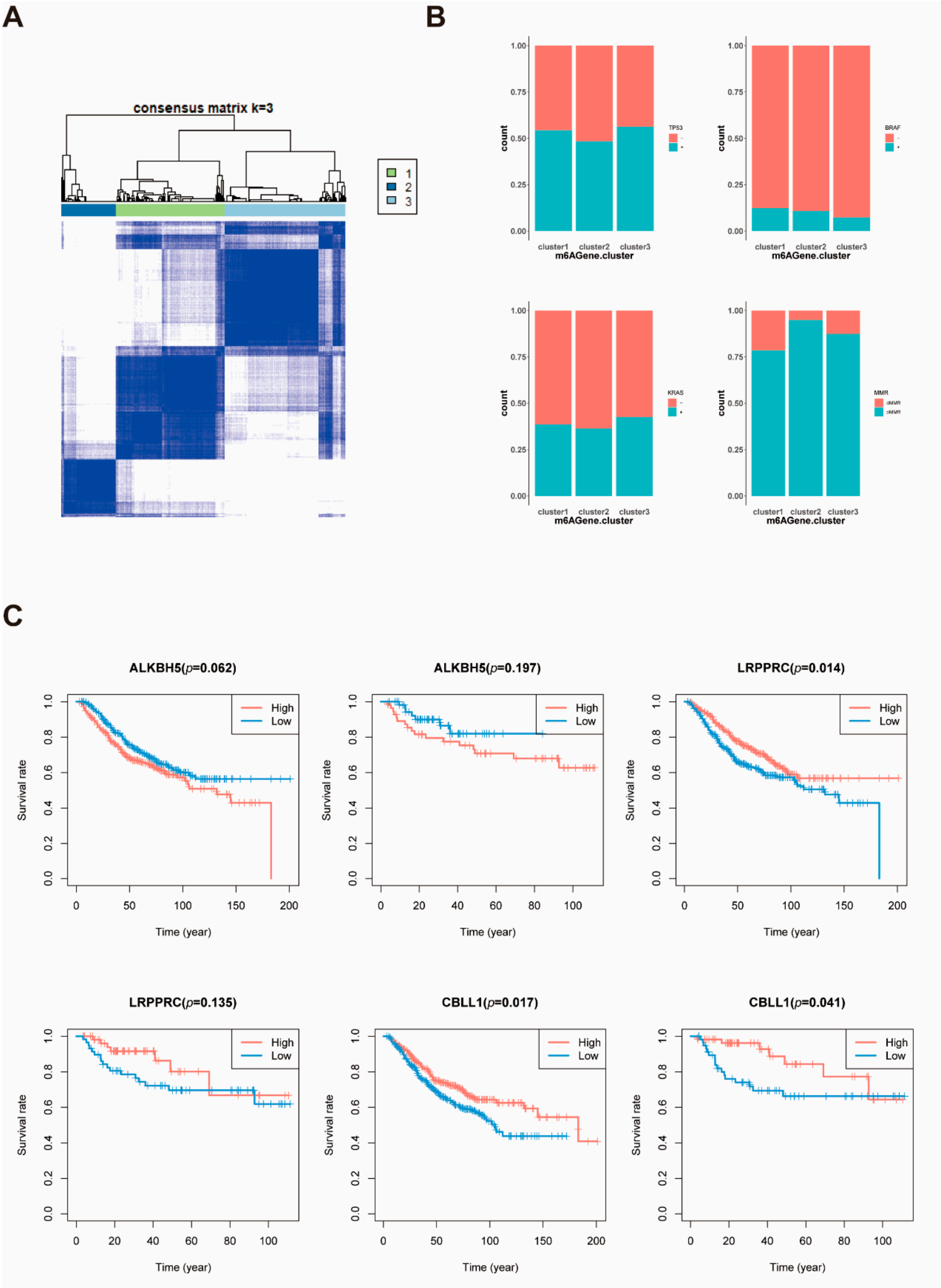

Figure S3. Characteristics of MMR-related genes, clinical features, and the roles of ALKBH5/LRPPRC/CBLL1 in colon cancer survival. (A) Unsupervised clustering of 299 m6A-related genes in GSE39582 and consensus matrices for k=3. (B) The expression of MMR-related genes in three m6A gene cluster. The upper and lower ends of the boxes represented interquartile range of values. The lines in the boxes represented median value, and the black dots showed outliers. The asterisks represented the statistical p value (*p<0.05, **p<0.01, ***p<0.001, ****p<0.0001). (C) Difference in the clinical features including BRAF/KRAS/TP53 mutant and MMR status among three m6A.cluster. (D) Survival analyses for patients with low or high ALKBH5/LRPPRC/CBLL1 expression in the GSE39582.

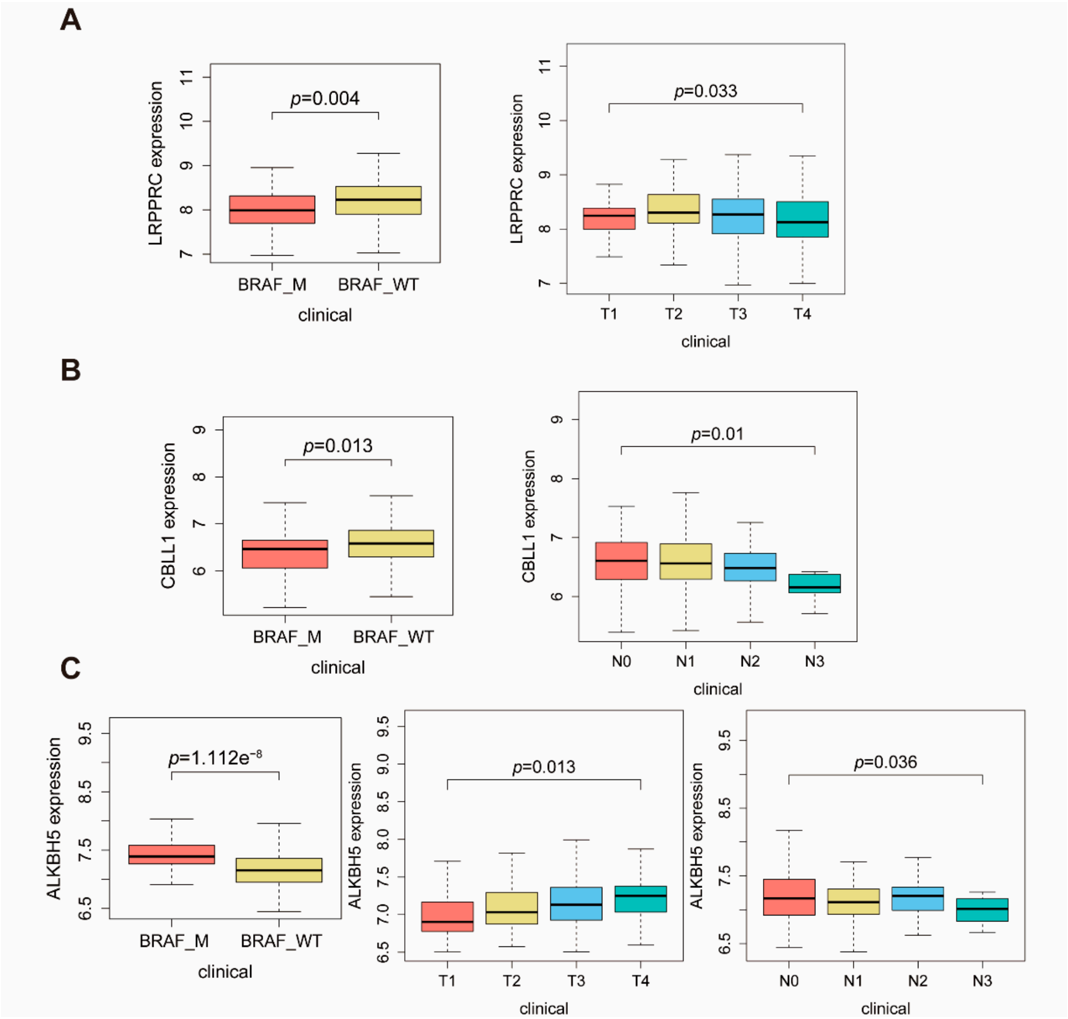

Figure S4. The relationship between ALKBH5/LRPPRC/CBLL1 and clinical information. (A) The expression of LRPPRC in BRAF+/- and T stage. (B) The expression of CBLL1 in BRAF+/- and N stage. (C) The expression of ALKBH5 in BRAF+/-, T stage and N stage.

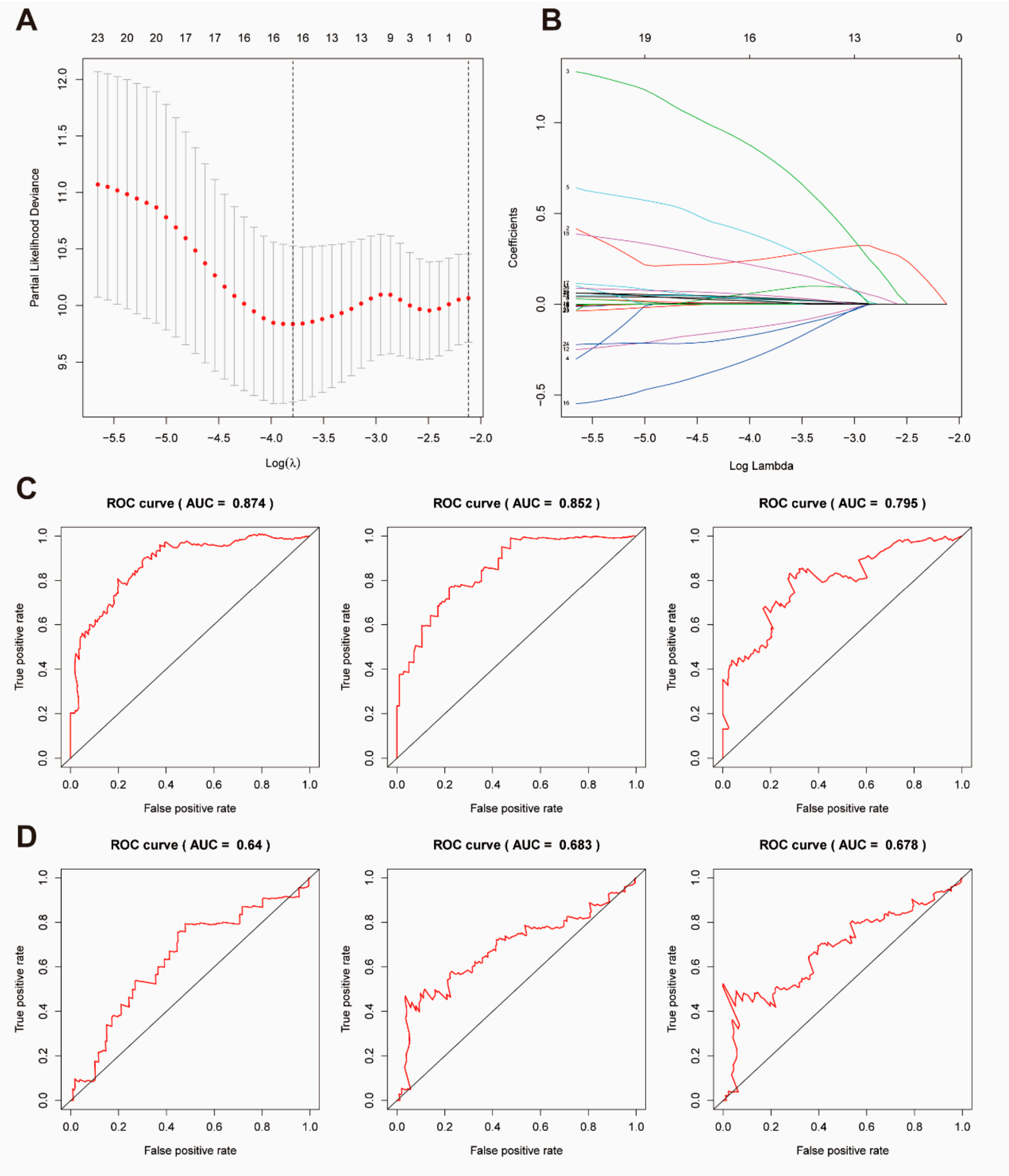

Figure S5. The results of Lasso regression and ROC curves of m6A risk score. (A) The results of cross-validation. The y-axis is partial likelihood deviance. The x-axis (bottom) is minimum. (B) The y-axis is the coefficient of the variables in the lasso regression equation. (C) ROC curve of the m6A risk score forecast result after 1,3, and 5 years in the training set. (D) ROC curve of the m6A risk score forecast result after 1,3, and 5 years in the test set.

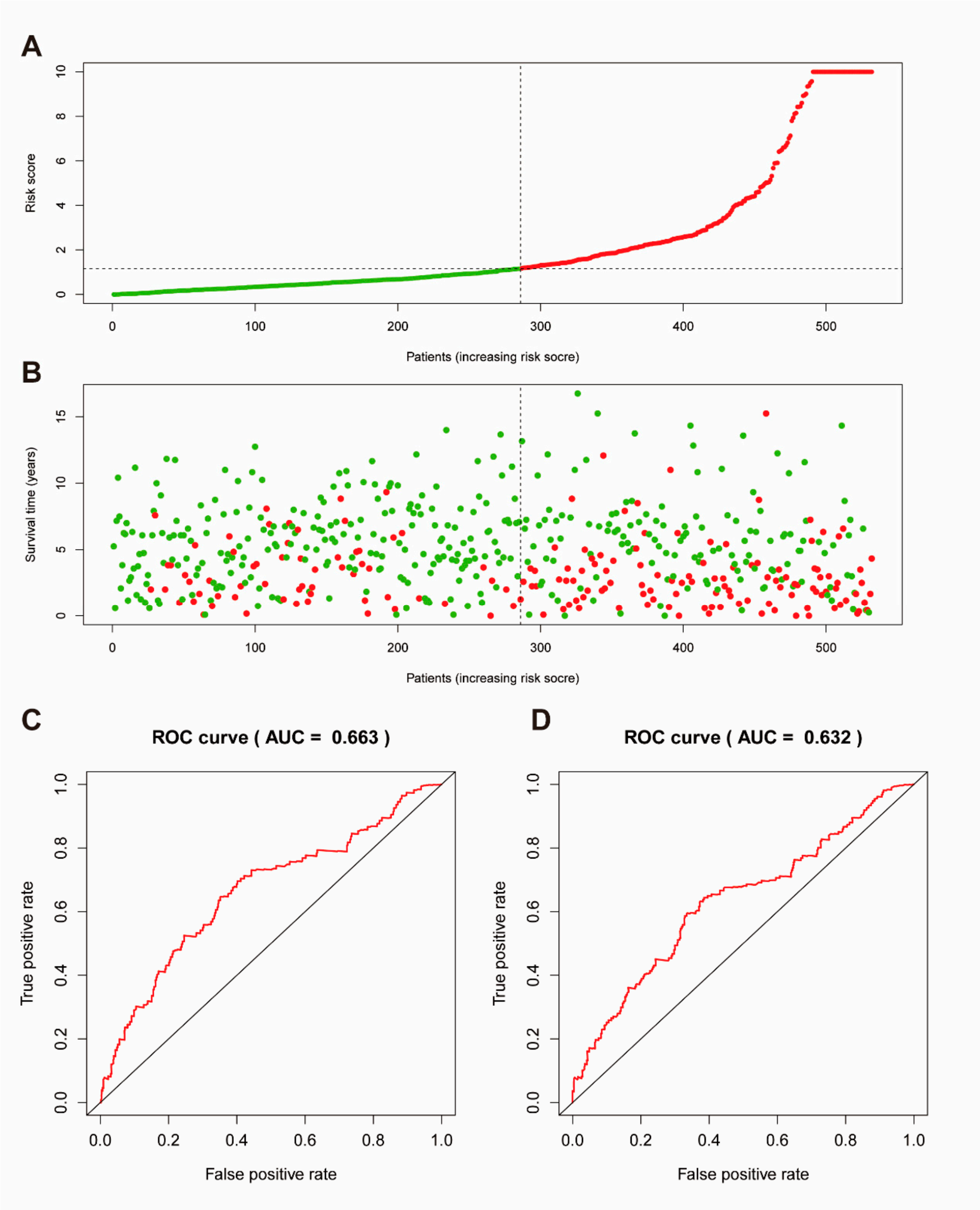

Figure S6. Validation of the prognostic performance of m6A risk score in GSE39582. (A) The distribution of risk score in GSE39582. The x-axis is the patient ranking in ascending order by the m6A risk score; the y-axis is the survival time. The red dots are the dead patients, and the green dots are the surviving patients. (B) The recurrence status in GSE39582. The x-axis is the patient ranking in ascending order by the m6A risk score; the y-axis is the survival time. The red dots are the dead patients, and the green dots are the surviving patients. (C-D) ROC curve of the m6A risk score forecast result after 3, and 5 years in GSE39582.

Table S1 Primer sequence corresponding to PrimerBank platform

Primer	Sequence 5′-3′
RBM15B-F	TACACGGAGGCTACCAGTACA
RBM15B-R	GTCGTACAGCCCGTAGTAGTC
ZC3H13-F	TCTGATAGCACATCCCGAAGA
ZC3H13-R	CAGCCAGTTACGGCACTGT
YTHDF1-F	ACCTGTCCAGCTATTACCCG
YTHDF1-R	TGGTGAGGTATGGAATCGGAG
CBLL1-F	TCCTTGGGTGGTCTTGATGTT
CBLL1-R	CAGGTTTCGCTTTGTTTGCTT
ELAVL1-F	GGGTGACATCGGGAGAACG
ELAVL1-R	CTGAACAGGCTTCGTAACTCAT
GAPDH-F	TGTGGGCATCAATGGATTTGG
GAPDH-R	TGTGGGCATCAATGGATTTGG

Table S2 Primer sequence corresponding to PrimerBank platform

siRNA	Sequence 5′-3′
Negative control-F	UUCUCCGAACGUGUCACGUTT
Negative control-R	ACGUGACACGUUCGGAGAATT
CBLLL-F	CACCAGAAAGCACCAUAUTT
CBLL1-R	AUAUGGUGCUUGUCUGGUGTT
